# The Role of Magnetic Resonance Imaging in Pre-operative Assessment of Anorectal Fistula With Surgical Correlation

**DOI:** 10.7759/cureus.53237

**Published:** 2024-01-30

**Authors:** Satyanarayana Kummari, Kiran Goud Burra, Vudem Ranjith Kumar Reddy, Saraswata Das, Rithika Ramadugu, Sameera Ramadugu

**Affiliations:** 1 Department of Radiodiagnosis, All India Institute of Medical Sciences, Nagpur, IND; 2 Department of Radiology, Government District Hospital, Medak, IND; 3 Department of Radiology, Government Medical College, Siddipet, IND; 4 Department of Radiodiagnosis, College of Medicine and JNM Hospital, Kalyani, IND; 5 General Practice, Kamineni Academy of Medical Sciences and Research Centre, Hyderabad, IND; 6 General Practice, Gandhi Medical College and Hospital, Hyderabad, IND

**Keywords:** suprasphincteric fistula, extrasphincteric fistula, transsphincteric fistula, anorectal fistulas, recurrent fistulas, secondary tracts, perianal abscess, intersphincteric fistula, mri, anal canal

## Abstract

Background

Anorectal fistulas are chronic inflammations of peri-anal tissues that form a connection between the perineal skin and the anal canal. Accurate preoperative evaluation of the main fistula's trajectory and pelvic tissues is essential for effective surgical treatment of anal fistulas. The inability to detect concealed lesions may result in the recurrence of fistulas and the conversion of a simple fistula into a complex fistula. Magnetic resonance imaging (MRI) imaging can detect concealed pathways and abscesses, thereby exposing the intricate connection between the fistula and anal-sphincter complex. This data serves as a roadmap for making surgical decisions, thereby reducing the likelihood of illness recurrence and complications after surgery.

Aim

To evaluate the role of MRI in pre-operative assessment of an anorectal fistula, compare its findings with surgical results.

Materials and methods

The research was conducted at the Radiology Department, Apollo Hospital, Hyderabad. It was a prospective observational study. IBM SPSS Statistics for Windows, Version 17 (released 2008; IBM Corp., Armonk, New York, United States) was utilized for data analysis. The mean and standard deviation were computed. We further applied appropriate statistical tests to determine the significance of MRI features with pre-operative findings.

Results

MRI accurately detects features like abscesses (sensitivity-100%, specificity-97.06%), secondary tracts of the fistula (sensitivity-93.55%, specificity-94.12%), horseshoe appearance, and supralevator extension (sensitivity-100%, specificity-97.50%, and 97.62%, respectively).

Conclusion

When comparing our results with intraoperative findings, MRI showed high sensitivity and specificity in detecting abscesses, secondary tracts, horseshoe appearances, and supralevator extensions. Our findings suggest that MRI can offer anatomical and pathological information for the pre-operative care and surgical planning of perianal fistulas.

## Introduction

A fistula is an unusual communication between the organs or between an inward organ and the skin. Anorectal fistulas are chronic inflammatory disorders affecting the peri-anal tissues that join the perineal skin and the anal canal [1]. These fistulas are more common in middle-aged men than in women, and the male-to-female ratio is 2:1 [2]. There are around 10 new instances for every 100,000 persons. In around 65% of cases, discharge is seen, and localized discomfort due to inflammation is also often reported [3]. It is believed that anal gland obstruction leads to the development of abscesses, which then burst through the skin to cause fistulas. However, they can also exist without showing any noticeable symptoms [4].

There are several potential causes of anal gland infection or fistula formation, including inflammatory bowel disorders, pelvic sepsis, diabetes mellitus, cancer, perineal trauma, and congenital factors [5].

Inflammation in the intersphincteric plane is common in cases of anal gland sepsis. It progresses along the intersphincteric plane to the perianal skin, a phenomenon found in approximately 70% of fistula cases. Another sort, transsphincteric fistulization, incorporates the infection going through the two layers of the butt-centric sphincter and the ischiorectal fossa; this occurs in around 20% of cases. Fistulous plots might bring about sore pits anyplace along their course; ischiorectal fossa abscesses are common in transsphincteric fistulas. Suprasphincter fistulization happens when infection sidesteps the sphincter complex and enters the ischiorectal fossa. Likewise, extrasphincteric or translevator fistulas might create assuming infection spreads from the pelvis to the skin through the ischiorectal fossa. Even after surgery that seems to be effective, there is a notable tendency for some fistulas to have recurrence rates of up to 25%. Often, the recurrences are the result of untreated infections from the original operation that were missed [6,7]. Depending on the procedure, the post-operative recurrence rate might reach 13.3%, with a median recovery period of 7.5 months [6,7]. Accurate preoperative examination of the pelvic tissues involved in the main fistula's route is essential for the successful surgical therapy of an anal fistula. The surgeon needs more than just an imaging diagnosis of the fistula. For the determination of the origin and anatomy of the fistula, detailed imaging is required. This data is essential for choosing the best surgical strategy and ensuring comprehensive care. Ignoring hidden lesions can lead to repeated fistulas, which can compound an otherwise simple case into a difficult fistulizing procedure. In difficult fistulizing procedures, there is a much lower likelihood of full recovery [6].

For a long time, the imaging method for anorectal fistulas was traditional fistulograms. Using this technique, the exterior opening is first accessed, and then the fistula is injected with a water-soluble contrast. There are two significant drawbacks to this strategy. First, when the primary tract and its extensions are blocked by pus or debris, the area is not completely visible because the area does not fill with contrast. Secondly, the sphincter muscle anatomy is not imaged [6]. Transrectal ultrasonography allows for better visualization of fistulae and their connection to the anal sphincter muscles. But it has drawbacks as well, like depending on operator competence, having a small field of view, and not having a coronal imaging plane [8].

Even though fistula in ano is not evaluated using computed tomography (CT), its poor soft tissue distinction and restricted imaging capability limit the accuracy of fistula classification. The attenuation values of the sphincter muscles, fibrotic areas, and fistula tract are comparable to each other, which limits the use of CT fistulography [8].

Milligan and Morgan first proposed the surgical classification of anal fistulae in 1934, and Stelzner and Goligher later modified it [9]. Park's widely accepted classification categorizes fistulae into four primary categories: intersphincteric, transsphincteric, suprasphincteric, and extrasphincteric, depending on their relation to the external sphincter [10,11].

The use of magnetic resonance imaging (MRI) as a diagnostic and preoperative assessment tool for fistula in ano has led to substantial breakthroughs in imaging methods, with MRI interpretations agreeing with surgical results in 14 of 16 instances [12,13]. Subsequent clinical observations corroborated the accuracy of MRI in finding fistulae, even in situations where examination under anesthesia (EUA) failed to detect distant infections [14]. It offers crucial insights into perianal fistulas' intricate anatomy [15-19].

The accuracy with which MRI shows the anatomy of the perianal area has been shown by Charles et al. [20]. This incorporates the butt-centric sphincter system and the connections among the fistulas, the pelvic floor, and the ischiorectal fossae [20].

With the utilization of MRI, concealed tracks and abscesses can be identified, and anatomical data about the fistula and the butt-centric sphincter complex can be gathered. To lower the risk of disease recurrence and surgical complications, this information is essential when making surgical decisions [21,22].

To our knowledge, there are only a few comprehensive studies in the literature evaluating the use of MR fistulography to thoroughly analyze fistulas in ano and compare the results with the surgical findings.

The primary objective of the research is to use MR fistulography to thoroughly analyze fistula in ano and compare the results to surgical findings so that surgical results are considered the gold standard. The sensitivity (Sn), specificity (Sp), positive predictive value (PPV), and negative predictive value (NPV) are assessed with the existence of supralevator extensions, abscesses, secondary tracts, and horseshoe fistulas.

## Materials and methods

The Apollo Hospital's Radiology Department in Jubilee Hills, Hyderabad, played host to this prospective observational research. Patients having a clinical diagnosis of perianal fistula were the primary focus of this 14-month research that was conducted between May 2017 and June 2018. The study cohort included 48 patients (34 males and 14 females) in the age range of 21 to 70 years. The Hospital Ethics Committee (HEC), Apollo Hospital, Jubilee Hills, Hyderabad, issued approval for the research, and the approval number was HEC/AHJ/2017/95.

Inclusion criteria

Patients with single or many discharging sinuses in the perianal region and those with recurrent perianal abscesses for undiagnosed tracks were included in the study.

Exclusion criteria

Patients with cardiac pacemakers, those with metallic implants in the body, foreign bodies in the eye, those with claustrophobia, and those under 21 years of age and beyond the age of 70 years were excluded from the study.

Study methods and procedure

The research was carried out from May 2017 to June 2018 on the patients who referred to the department of radiology for MR fistulograms. Informed written consent was obtained from all the patients. The patient was positioned laterally, the external fistula hole was located and cannulated, and saline was injected. After that, the MR gantry was moved such that the subjects were lying supine for imaging.

Research imaging methods

Patients were not asked to change into any special attire for their MRI, which was performed using a 1.5 Tesla Philips machine (Koninklijke Philips N.V., Amsterdam, Netherlands) with a phased array coil. To design the ensuing coronal, axial, and sagittal views, a scout sagittal slice was first taken via the anorectal area. The following sequences were taken, stretching from the perianal area to the level of the levator ani muscle. Various sequences were used, and their parameters are listed in Table 1.

**Table 1:** Various sequences made use of the following parameters STIR: short tau inversion recovery; SPAIR: spectral attenuated inversion recovery; TSE: turbo spin echo; FFE: fast field echo; TR: repetition time; TE: time to echo; FOV: field of view

Parameters	STIR	T2 SPAIR	T2 SPAIR	T2 SPAIR	T2 TSE	T2 TSE	T2 TSE	T2 FFE	T1 TSE
Imaging plane	Sagittal	Sagittal	Coronal	Axial	Sagittal	Coronal	Axial	Axial	Axial
TR/TE (m sec)	4500/75	2850/100	Shortest/80	2850/100	4000/90	4000/100	4000/100	650/13	500/10
FOV (mm)	240x260x133	220x220x90	220x220x140	240x258x174	220x220x90	220x220x140	240x258x174	240x258x174	240x258x174
Intersection gap (mm)	0.30 mm	0	0	0	0	0	0.81 mm	0	0
Section thickness	3.5 mm	3.0 mm	3.5 mm	3.5 mm	3.5 mm	3.5 mm	2.6 mm	3.5 mm	3.5 mm
Matrix	148x124	232x196	232x188	184x130	232x188	244x226	268x263	184x101	244x211

Classification of parks

Intersphincteric

The fistula crosses the intersphincteric space and does not cross the external sphincter.

Transsphincteric

The fistula spreads from the intersphincteric space, over the external sphincter, and into the ischiorectal fossa.

Suprasphincteric

The fistula ascends into the intersphincteric region and, afterward, passes across the puborectalis muscle and into the ischiorectal fossa through the iliococcygeus muscle.

Extrasphincteric

The fistula starts outside the outer butt-centric sphincter and stretches out from the perineal skin, past the ischiorectal fossa and the levator ani muscle complex, and into the rectum.

Sorts of emergency clinics at St. James' College: Grade 1: Direct intersphincteric stoma; Grade 2: Sore or optional track portrays an intersphincteric ulcer; Grade 3: Transsphincteric; Grade 4: Boil or optional track inside the ischiorectal fossa describes a transsphincteric condition; Grade 5: Supra- and trans-levator extension [12].

Learn about statistical techniques

The surgical results were used as the gold standard against which the MRI findings were compared. The MRI was considered accurate if it properly classified and localized the principal track, abscess, horseshoe component, and subsidiary tracts, as seen during the concluding surgical procedure.

Review, coding, tabulation, and analysis were performed on the collected data with the help of a biostatistician using IBM SPSS Statistics for Windows, Version 17 (released 2008; IBM Corp., Armonk, New York, United States). Quantitative information was displayed as means and standard deviations (SD), while subjective information was displayed as frequencies and rates. When fitting, we utilized factual tests, including the one-way Chi-Square, and we respected results as being measurably critical when p < 0.05.

A factual examination was performed to gauge the viability of MRI in identifying perianal fistulas in light of the last careful results. Sensitivity (accuracy in positive cases), specificity (accuracy in negative cases), overall accuracy, positive predictive value (accuracy in positive results), and positive predictive value (accuracy in negative results) were calculated and analyzed to determine how well MRI could predict the presence of abscesses, secondary tracts, horseshoe appearances, and supralevator extensions.

True positive (Tp) (surgery +, MRI +), false positive (Fp) (surgery -, MRI +), false negative (Fn) (surgery +, MRI -), and true negative (Tn) (surgery -, MRI -) cases were assessed.

## Results

The information collected consists of both demographic facts and variables associated with a certain medical condition. Of the 48 participants, women made up just 29.2% and men 70.8%. Participants' ages ranged from 21 to 70 years. Regarding fistula types, 58.3% were primary cases, and 41.7% were recurrent. Noteworthy high-risk factors included Crohn's disease (25%), diabetes (25%), the absence of risk factors (35%), and tuberculosis (15%). Importantly, the research found relationships between gender and fistula type, pointing to possible links within the medical condition. Statistics and clinical characteristics of patients with anorectal fistulas (Table 2).

**Table 2:** Statistics and clinical characteristics of patients with anorectal fistula DM: diabetes mellitus; TB: tuberculosis

		Frequency (n)	Percentage (%)	P value
Gender	Female	14	29.2	0.0036
	Male	34	70.8	
Age distribution	21-30	9	18.8	0.2190
	31-40	9	18.8	
	41-50	16	33.3	
	51-60	7	14.6	
	61-70	7	14.6	
Fistula type	Primary	28	58.3	0.2676
	Recurrent	20	41.7	
High-risk factors for recurrence	Crohn’s	5	25	0.6590
	DM	5	25	
	None	7	35	
	TB	3	15	

The analysis demonstrates a strong correlation between the presence of an abscess and MRI grades. In particular, 12.50 percent of Grade 2 fistulas showed the prevalence of abscesses, compared to 10.40 percent and 8.30 percent for Grade 3 and Grade 4 fistulas, respectively. Among these grades, the correlation between the presence of an abscess and a Grade 2 fistula was statistically significant, as indicated by a low p-value of 0.004.

Similarly, there was a clear association found between the presence of secondary tract and MRI grades. In particular, in Grade 2 fistulas, the incidence was 12.50%, Grade 4 was 16.70%, and Grade 5 was 8.30%. The correlation between the presence of secondary tracts and Grade 2, 4, and 5 fistulas was statistically significant, as indicated by a low p-value of 0.0007.

Even though there seems to be a link between greater prevalence and higher MRI grades of the fistulas and the horseshoe component, the higher p-value of 0.06 indicates that this association is not statistically significant. Characteristics of the MRI in patients with anorectal fistulas (Table 3).

**Table 3:** Characteristics of the MRI in patients with anorectal fistulas MRI: magnetic resonance imaging

		MRI Grade										P value
		1		2		3		4		5		
		Cases	%	Cases	%	Cases	%	Cases	%	Cases	%	0.004
Abscess	Yes	0	0.0	6	12.5	0	0.0	5	10.4	4	8.3	
	No	14	29.2	4	8.3	5	10.4	7	14.6	3	6.2	
Total		14	29.2	10	20.8	5	10.4	12	25.0	7	14.6	
Secondary tract	Yes	0	0.0	6	12.5	0	0.0	8	16.7	4	8.3	0.0007
	No	14	29.2	4	8.3	5	10.4	4	8.3	3	6.2	
Total		14	29.2	10	20.8	5	10.4	12	25.0	7	14.6	
Horseshoe component	Yes	0	0.0	2	4.2	0	0.0	4	8.3	3	6.2	0.06
	No	14	29.2	8	16.6	5	10.4	8	16.7	4	8.3	
Total		14	29.2	10	20.8	5	10.4	12	25.0	7	14.6	

Fifteen patients had their abscesses correctly detected by MRI; all of them had surgery, indicating a strong link. Similarly, 16 out of 18 cases with secondary tracts identified by MRI required surgery. In eight of the nine cases, an MRI showing a horseshoe shape led to surgery; in the other 39 patients, no surgery was necessary. Six surgeries were necessary as a result of supralevator extension found in seven patients, emphasizing the critical role MRI plays in pointing surgeries. Comparison of the MRI findings of anorectal fistulas with surgical findings (Table 4).

**Table 4:** Comparison of the MRI findings of anorectal fistulas with surgical findings MRI: magnetic resonance imaging

MRI		Surgery		Total
		Yes	No	
Abscess	Yes	14	1	15
	No	0	33	33
Secondary tract	Yes	16	2	18
	No	1	29	28
Horseshoe appearance	Yes	8	1	9
	No	0	39	39
Supralevator extension	Yes	6	1	7
	No	0	41	41

When compared to the gold standard, MRI's diagnostic accuracy in identifying abscesses is remarkable, with a sensitivity of 100% and a specificity of 97.06%. Sensitivity (93.55%) and specificity (94.12%) for locating secondary tracts are likewise very good. MRI has a perfect sensitivity (100%) and a high specificity (97.50% and 97.62%, separately) when contrasted with the best quality level for recognizing horseshoe appearance and supralevator extension. Various results highlight the value of MRI as a robust technique for reliably detecting various diseases. Comparison of sensitivity, specificity, PPV, and NPV of MRI with surgery (Table 5).

**Table 5:** Comparison of sensitivity, specificity, PPV, and NPV of MRI with surgery Sn: sensitivity; Sp: specificity; PPV: positive predictive value; NPV: negative predictive value; CI: confidence interval

	Abscess		Secondary tract		Horseshoe appearance		Supralevator extension	
	Value	95% CI	Value	95% CI	Value	95% CI	Value	95% CI
Sn	100	76.84- 100	93.55	78.58-99.21	100	63.06-100	100	54.07-100
Sp	97.06	84.67-99.93	94.12	71.31-99.85	97.50	86.84-99.94	97.62	87.40-99.64
PPV	93.33	67.00-98.97	96.67	81.21-99.49	88.89	53.60-98.23	85.71	46.39-97.65
NPV	100	-	88.89	67.56-96.85	100	-	100	-
Accuracy	97.92	88.93-99.95	93.75	82.80-98.69	97.92	88.93-99.95	97.92	88.93-99.95

## Discussion

**Figure 1:**
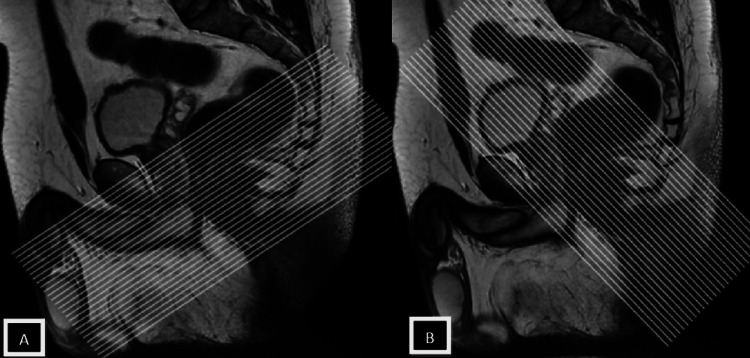
Sagittal T2-weighted midline images Sagittal T2-weighted midline images were employed in axial MR imaging of the anal canal to guarantee accurate axial alignment. For a coronal MRI, pictures were taken opposite the long hub of the butt-centric waterway, at the right point to the pivotal plane.

MR fistulography, which uses high-resolution MRI to provide a thorough insight into the anal sphincter architecture, has been demonstrated to be helpful in the preoperative assessment of perianal fistulae. The parameters utilized for each MRI sequence are illustrated in Table 1. The suggested orientation for axial MR imaging of the anal canal is depicted in Figure 1.

In the present research, MR fistulography was conducted on 48 cases diagnosed with perianal fistula, aged between 21 and 70 years. Among the participants, 70.8% were males, with a mean age of 44, and the most commonly affected age group was 41-50 years (33.3%) (Table 2). One possible explanation for why males are more affected is because they have more anal glands, which are typically more cystic and complex compared to females. This observation is in line with a study by N.Mohey et al. that also noted a similar male preponderance, with 96.7% males in their patient cohort [23]. Similar observations were noted in a study conducted by Mohamed RE et al., in which 66.6% of patients were males [24].

In this analysis, we divided instances into two classes: primary and recurring. The major group consisted of persons with fistulas in ano for the first time. In contrast, patients in the recurrent group had previously undergone at least one operation for their fistulae. Primary fistulas were seen in the majority of patients (28, or 58.3%) in our analysis. Out of 56 patients with butt-centric fistulas, 24 had starting fistulas and 17 had repeating fistulas, as was displayed in an exploration by Beets-Tan et al. [25]. Preoperative magnetic resonance imaging fistulography for patients with perianal fistulas was evaluated in another study by Waniczek D. et al. [1]. There were a total of 14 participants in the study; eight had initial fistulas, and six had recurring fistulas.

Twenty individuals with recurring fistulas were examined, and five were found to have Crohn's disease, five to have diabetes mellitus, and three to have TB. The importance of these variables on the morbidity of fistula in ano, especially its recurrence, was highlighted by the fact that 65% of recurring cases were linked to risk factors. Beets-Tan et al. found that 12 of 56 patients with anal fistulas had Crohn's disease as a risk factor [25], hence the two studies seem to confirm each other. In addition, research by Khera PS et al. found that Crohn's disease was present in three of 35 instances with a primary or recurrent perianal fistula [17].

**Figure 2:**
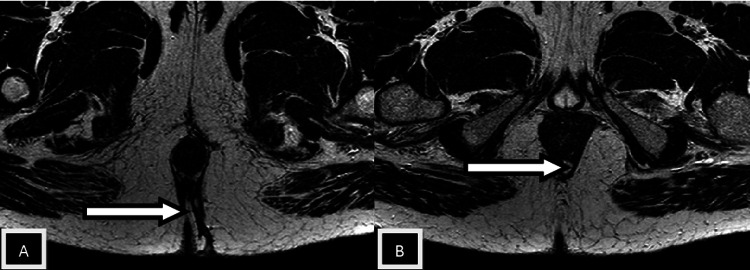
Axial T2w images Images showing an intersphincteric tract (white arrow in image A) with an intersphincteric abscess (white arrow in image B)

**Figure 3:**
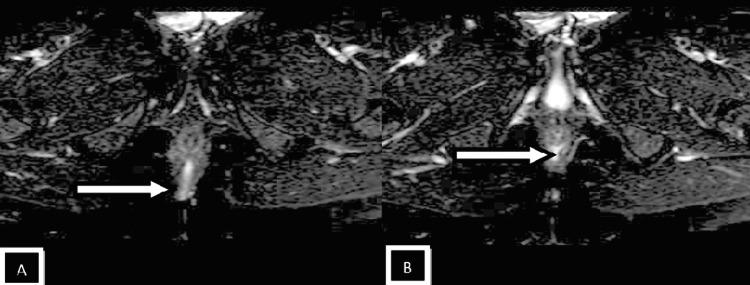
Axial SPAIR images Images showing the intersphincteric tract penetrating the external sphincter at the 6 o'clock position (white arrow in images A and B) SPAIR: spectral attenuated inversion recovery

**Figure 4:**
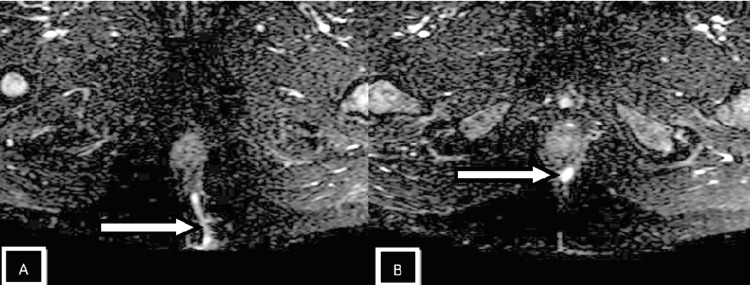
Axial SPAIR images Images showing an intersphincteric tract (white arrow in image A) with an intersphincteric abscess (white arrow in image B) SPAIR: spectral attenuated inversion recovery

**Figure 5:**
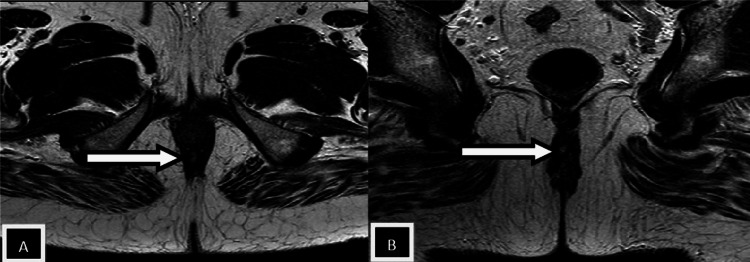
Axial and coronal T2W images Images showing a non-branched, transsphincteric fistulous tract with an internal opening at the 6 o'clock position

**Figure 6:**
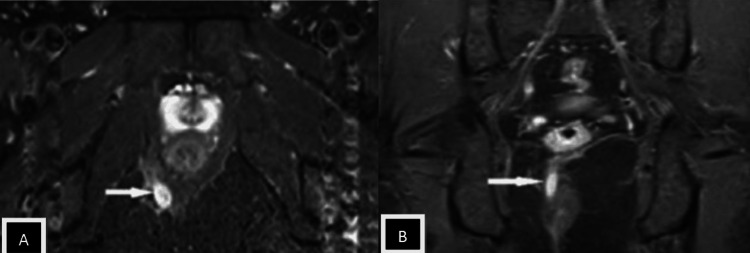
Axial and coronal STIR images Images showing a fistulous tract (white arrow in image A), which has crossed the external sphincter and lying in the right ischio-rectal fossa (white arrow in image B) STIR: short tau inversion recovery

Patients with perianal infection who had a medical procedure were grouped by the St. James' College Emergency Clinic Arrangement. In light of radiologically conspicuous elements in the coronal and axial planes, this classification framework recognizes essential and auxiliary fistulous tracts and related abscesses. Patients with intersphincteric fistulas of Grades 1 and 2 (simple linear) and 3 and 4 (intersphincteric with abscess/secondary track) were combined because of a lack of data. Grade 5 fistulas (supralevator and translevator diseases) were categorized separately [12,13]. In the current study, intersphincteric fistulas (Grades 1 and 2) (Figures 2, 3, 4) were most common, found in 24 patients (50%), followed by transsphincteric (Grades 3 and 4) (Figure 5) in 17 patients (35.4%), and suprasphincteric fistulas (Figure 6) in seven patients (14.6%) (Table 3). Similar findings were observed in related studies: Mohamed RE et al. reported Grade 1 as the most common type in nine patients (37.5%) [24].

In the present study, the most common internal fistulous opening (clock position) was 6 o'clock in 24 subjects (48%) (Figures 2, 3, 4, 5). The results are similar to Mohamed RE et al., where 12 (50%) of cases had an internal fistulous opening at the 6 o'clock position [24].

In our study, MRI demonstrated remarkable accuracy in diagnosing perianal fistulas. Secondary tract detection exhibited a sensitivity of 93.5% and a specificity of 94.12%, while canker determination gave 100 percent responsiveness and 97.06% explicitness. MRI excelled at identifying horseshoe appearances with 100% sensitivity and 97.5% specificity. Overall, it exhibited accuracy rates of 93.75% for secondary tract detection, 97.92% for abscess identification, and 97.9% for horseshoe tract detection (Table 4, 5). These findings align with Villa C et al.'s confirming MRI's crucial role in precise perianal fistula assessment, ensuring effective management and reduced complications [26]. Similarly, Beets-Tan et al. studied pre-operative MR imaging of anal fistulas in 56 patients. This included primary and recurring fistulas caused by Crohn's disease. Detection rates for sensitivity and specificity were 100% and 86% for fistulous tracts, 96% and 97% for abscesses, 100% and 100% for horseshoe fistulas, and 96% and 90% for internal openings [25]. The sensitivity of MRI for grading primary perianal fistulas was 95.8%, specificity was 83.3%, PPV was 95.8%, and NPV was 83.3%, with a P-value of 0.000, according to a study conducted by N.Mohey et al. [23].

MRI is a highly effective and reliable preoperative imaging method for perianal fistulas. Its critical function is to precisely determine the extent of the disease and how it interacts with the sphincter complex. Through the identification of secondary extensions, such as abscesses and horseshoe tracts, MRI provides a thorough assessment, offering the highest level of diagnostic precision. This comprehensive information is essential for effective surgical procedures that reduce complications and the chance of recurrences, improving overall management results.

There are a few limitations inherent in our research. A limited sample size of patients from a single institution was studied. It was a short-duration study. We did not include patients under 21 years of age and beyond the age of 70 in our study. However, the implementation of more comprehensive research endeavors with a larger cohort would yield a more precise depiction of the matter under investigation.

## Conclusions

In this study, primary anorectal fistulas were more common (58.3%) than recurrent ones. The internal opening is noted at the 6 o'clock position in half of the examples. Grade 4 fistulas had more secondary tracts, whereas Grade 2 fistulas had numerous abscesses. When comparing our results with intraoperative findings, MRI showed high sensitivity and specificity in detecting abscesses, secondary tracts, horseshoe appearances, and supralevator extensions. Our findings suggest that MRI can offer anatomical and pathological information for the pre-operative care and surgical planning of perianal fistulas. However, the study recommends further validation through a larger sample size and long-duration studies. Conducting a multicenter study would contribute to a more comprehensive validation of the study's results.
